# “There is no need to leave the beach to test”: A qualitative study of HIV self-testing knowledge and acceptability of HIV self-test kit distribution among social networks of fishermen in western Kenya

**DOI:** 10.21203/rs.3.rs-5090648/v1

**Published:** 2024-11-27

**Authors:** Jayne Lewis-Kulzer, Phoebe Olugo, Sarah A. Gutin, Zachary A. Kwena, Holly Nishimura, Marguerite Thorp, Kawango Agot, Benard Ayieko, Elizabeth A. Bukusi, Lennah Oluoch, David Angawa, Harsha Thirumurthy, Carol S. Camlin

**Affiliations:** University of California San Francisco; Impact Research and Development Organization; University of California San Francisco; Kenya Medical Research Institute; University of California San Francisco; University of California Los Angeles; Impact Research and Development Organization; Impact Research and Development Organization; Kenya Medical Research Institute; Impact Research and Development Organization; Impact Research and Development Organization; University of Pennsylvania; University of California San Francisco

**Keywords:** HIV self-testing, prevention, fishermen, peer approaches, sub-Saharan Africa, Kenya

## Abstract

**Background:**

HIV self-testing (HIVST) can improve HIV testing uptake by offering convenience and privacy. Yet HIVST accessibility and uptake remain limited in Lake Victorias beach communities where HIVST holds promise to address many barriers highly mobile populations of men in fishing communities face. We assessed HIVST knowledge and acceptability among highly mobile fishermen, a high priority population for HIV prevention and treatment, participating in a social network-based study (“Owete”; NCT04772469) to promote HIV testing, prevention, and treatment in Kenya.

**Methods:**

Sixty-five in-depth baseline interviews (IDIs) and two focus group discussions (FGDs) were conducted at study baseline from December 2021 to June 2022 with fishermen, including 30 who were social network-central men recruited as HIVST “promoters” from three fishing communities along Lake Victoria, Kenya. Fishermen were purposively-sampled based on study arm, community and age (18–34 and 35+) for interviews exploring HIVST knowledge, perceived benefits, and concerns. IDIs and FGDs were audio-recorded, translated/transcribed into English and inductively-coded and analyzed by six researchers using a framework approach.

**Results:**

Nearly all participants had heard about HIVST and expressed willingness to self-test. Almost half reported learning about HIVST for the first time through the Owete study. Perceived benefits of self-testing included privacy, convenience, and being able to learn one’s status with the freedom to choose when and where to test, which minimized stigma and work interruptions. Few participants had used HIVST prior to joining Owete, all of whom reported ease of use. Potential barriers to HIVST included fear of HIV-seropositive results, feeling unsure about how to use HIVSTs, and fear of stigma if a HIVST was discovered. Nearly all Owete promoters indicated willingness to distribute HIVST to help their peers know their status. Promoters stressed the importance of approaching HIVST discussions strategically and thoughtfully to garner trust and engagement, and felt they needed training to answer HIVST questions.

**Conclusion:**

While few fishermen had ever used HIVST, this study found high awareness, positive perceptions, and substantial willingness to use and distribute HIVST to other men. The “promoter” model, with known peers engaged in disseminating HIVST information and test kits, shows promise for engaging men in testing.

## INTRODUCTION

While sub-Saharan African (SSA) nations have made great progress towards the Joint United Nations Programme on HIV/AIDS (UNAIDS) 95-95-95 targets, men’s engagement in HIV testing, prevention, and treatment remains suboptimal in many settings in the region. Twenty-one percent of men living with HIV in SSA in 2020 were unaware of their HIV status (1, 2). Learning one’s HIV status through testing is the first step in the cascade to HIV prevention and treatment (3). Yet, in SSA men lag substantially behind women in HIV testing (4, 5). In Kenya, in 2018, a significantly lower proportion of men had ever tested for HIV compared to women (70.7% men vs. 85.1% women) and fewer men than women know their HIV status (72.6% men vs. 82.7% women) (5). Lower HIV testing among men hinders their ability to access HIV prevention, treatment and to become virally surpressed.

Men face many barriers to facility-based HIV testing including difficulty accessing HIV testing services at fixed location clinics because of work-related travel or work schedules that make clinic attendance difficult (6-9). These barriers can be exacerbated by norms of masculinity that value strength and see HIV testing or seeking care as weakness (6, 10, 11) and by stigma associated with clinic attendance (8, 12, 13). In addition, men may view clinics as female spaces where the focus is on women’s reproductive health and infant and child health (4, 14, 15). Men have also been found to infer their HIV status from their female partners who have tested (16).

The Lake Victoria region has the highest HIV prevalence in Kenya, ranging from 9.9–19.6% (compared to a national adult HIV prevalence of 4.9%) and 36,000 new cases annually in 2018 (5). Fisherfolk living around Lake Victoria are a priority population contributing to the high HIV prevalence in the region and in need of improved HIV testing, prevention, and care services (17, 18). The highly mobile lifestyle of fishermen moving from beach to beach in search of good fish (6, 9), their engagement in higher-risk sexual behaviors, including transactional “sex for fish” economy embedded in the fishing trade (19-24) and poor HIV care engagement (6, 8, 9) are factors that contribute to the higher HIV prevalence.

HIV self-testing is a new approach that may be preferrable for fishermen and other priority populations because it is convenient, can be conducted at a time and place of the user’s choice, and results can be kept private (25-27). In SSA, various approaches for distribution of HIV self-testing kits for men have shown promise, including through peers (28-30), female partners (31) and community-based distributors (32-36). Research using social network influence to distribute HIV self-testing kits among highly mobile fishermen has not been previously studied. In the Owete Study, social network-central male peers were trained to distribute HIV self-testing kits to men in their close social networks. In this baseline qualitative study, we assessed HIV self-testing knowledge and acceptability among highly mobile fishermen in communities along Lake Victoria, Kenya.

## METHODS

### Parent study

This qualitative study was embedded in a large cluster randomized controlled trial, the “Owete” (“Brothers” in Dholuo) study (NCT#04772469)(37). The Owete study evaluated the effectiveness of a HIV status-neutral, social network-based approach combining behavioral and biomedical strategies to increase HIV testing uptake and linkage and adherence to prevention and care services among highly mobile male fishermen (37). The study team conducted a census of men working in the fishing industry in three beach communities in Siaya County using Beach Management Unit registries of fishermen. The study then conducted a full sociometric survey of the male social networks of men in the registries. Network-central men (“promoters”) and their close social network clusters were then randomized in clusters to intervention or control arms. Promoters in both arms participated in a one-day of basic training on HIV prevention and treatment, while promoters in the intervention arm participated in a second day of training HIV self-testing kit use and interactive sessions on peer communication and linkage to facilities. After promoter training, promoters in the control arm received non-monetary referral vouchers to distribute to their social network members for routine HIV testing at a health facility. Promoters in the intervention arm received HIVST kits for distribution to their social network members, along with incentive vouchers for Kenyan Shilling (KSHS) 500 (about US Dollar [USD] $3.50) to facilitate health facility linkage following HIV self-testing use.

### Study setting and population

The Owete study took place in Siaya County along the shores of Lake Victoria in western Kenya. This setting was ideal due to its prominence as a hub for fishing activities, supporting a population of 993,183 individuals as of 2019 (38). The county exhibited an HIV prevalence rate of 15.3%, surpassing the national average of 4.9%, with HIV testing and viral suppression higher than national levels yet below the UNAIDS 95-95-95 goals: in 2018, HIV testing in the last 12 months was 70.4% in Siaya and 43.6% nationally and viral suppression was 78.7% in Siaya and 71.6% nationally (39, 40).

### Sampling and recruitment

Qualitative study participants were purposively sampled from the population of men participating in the Owete trial, selected for heterogeneity across the groupings of age (18–34 and 35+), beach community, study arm, and promoter versus social network cluster member categories. The selected fishermen were recruited from December 2021 to June 2022. Research team members trained in qualitative research techniques conducted a total of 65 in-depth interviews (IDIs) and two focus group discussions (FGDs) comprising 8–12 participants each.

### Data collection

IDIs and FGDs were carried out by four trained research assistants proficient in the participants' preferred languages, Dholuo or Swahili. The IDIs and FGDs were conducted between December 2021 and June 2022, within a month of the main trial’s enrollment consenting process and quantitative baseline survey administration at each beach. The consent process and survey introduced the HIV self-testing, however no community or peer sensitization or distribution of HIV self-testing was carried out prior the baseline IDIs and FGDs. IDI and FGD guides explored participants’ understanding, perceived advantages, and concerns regarding HIV self-testing. We asked promoter participants about their willingness to distribute test kits and encourage HIV self-testing uptake and linkage to care or prevention within their respective groups. Each interview lasted approximately one and a half hours. Participants were compensated KSHS 500 (approximately $3.50 USD) for their time.

### Data analysis

IDIs and FGDs were translated/transcribed from either Dholuo or Swahili into English. An extensive coding and analysis process was undertaken by a team of six researchers using both inductive and deductive approaches, following a framework methodology (41). This involved developing a coding framework by combining deductive methods, drawing codes from theory-informed interview guides, and inductive methods, identifying potential inductive codes from Charmaz’s two-stage process involving line by line open coding of initial transcripts followed by development of focused codes (42). The codebook underwent refinement as needed through additional data review and an inter-coder reliability process among the six coders. The six coders, including a senior Kenyan social scientist, a Kenyan qualitative research coordinator, and four qualitative research scientists based in the United States (U.S.), collaborated after coding approximately two transcripts each to ensure consistent code application. Any discrepancies among the coders were addressed through discussion to reach consensus, periodically refining code definitions. Codes were applied using Dedoose software (43). After the initial coding, a team-based framework analysis approach, led by a senior U.S.-based investigator, was employed to collaboratively identify and organize major and minor emergent themes in the data that were salient to the key objectives of the study to understand facilitators and barriers to HIV self-testing usage among social network cluster members, and promters’ willingness to distribute and promote self-testing among their peers (41).

#### Ethical approval

The research obtained approval from Kenya Medical Research Institute-Scientific Ethics Review Unit (KEMRI-SERU; #677) and the University of California, San Francisco - Institutional Review Board (UCSF-IRB; #19-20285). Prior to involvement in the study, all participants provided written informed consent.

## RESULTS

Among the 65 fishermen in this qualitative sub-study, about half of the men were under 35 years of age. Most men (83%) were married and had either attended some or completed primary school (78%), while the remaining (22%) had completed secondary school. Of the three beaches in Siaya County where this study took place, beach 1 had slightly fewer participants than beaches 2 and 3. When asked about income, nearly two-thirds of men reported earning less than 10,000 KSHS/80 USD per month (Table 1).

In the results below, we describe participants’ knowledge of HIVST and their source of information, their experiences with and perceptions of HIV self-testing, including willingness to use HIVST, the perceived benefits of self-testing, and the challenges they anticipate with using HIVST. Lastly, we describe promoters’ reported willingness to distribute HIV self-testing kits to those within their close social networks.

### Fishermen’s knowledge of HIV self-testing

Knowledge of HIV self-testing was close to universal, with nearly all participants saying they had heard about HIV self-testing, with approximately half reported that they had first learned about HIV self-testing through the Owete study, i.e. shortly before the interview. The Owete study had described HIV self-testing during the informed consent process since it was a key component of the planned intervention and during a separate quantitative survey. A participant explained, “[I heard about HIV self-testing] from the research team, they were the first ones to introduce us to them [HIV self-testing].” *Beach 3: <35 years of age*. Other sources of HIV self-testing information included health facility staff, community health workers, and advertisements. A participant from Beach 3 said: “When I go to the hospital I’m normally told if you fear testing, I can give you this thing to carry to the house and test yourself.” *Beach 3; <35 years of age*. Several participants understood that the self-testing process involves swabbing gums for saliva for an immediate result, as this participant explained: “…now there’s some gadget which is swabbed on the gums. You swipe it in the gum this way then there is some liquid that you add to it, then you wait for some time, then the results will be out.” *Beach 3; <35 years of age*. Men were also aware that it can be done anywhere and anytime. Some participants mentioned HIV self-testing availability at health facilities and chemists, but few had actually seen a HIV self-testing kit.

In Kenya, once a person has used an HIV self-testing kit, a formal HIV test at health facility is advised to confirm results. In this sample, only one participant mentioned the need for confirmatory testing at the health facility following testing via HIV self-testing.

### HIV self-testing experience, willingness to use, and perceived benefits

While few (n=7) participants had used HIV self-testing prior to joining Owete, nearly all recognized the importance of knowing their HIV status, expressed willingness to use HIV self-testing, and had a positive outlook on its utility. Findings revealed that men identified privacy and convenience as the most pronounced benefit of self- testing compared to facility-based testing, as illustrated in the following quote: “It [HIV self-testing] can be used anytime …and you can use them when you are alone.” *Beach 3; <35 years of age*. Knowing that one can test at home away from others’ eyes was viewed as an important feature to prevent stigma, gossip and inadvertent disclosure, as expressed by this participant:“It [HIV self-testing] is good because nobody gets to know your secret since you use it on your own and you are the one who gets to know your status alone.” *Beach 2; ≥35 years of age*. The freedom to choose when and where to test was seen as a valuable perk, by making testing easier, more accessible, and not distrupting work schedules by needing to go to a healthy facility to test, as described by this participant: “If it is something easier for me to use, I would just use it because you find that the hospital is at distance and you are busy at work. So when I have it in that case, I can test at any time. So that I can get to know my status.” *Beach 1; <35 years of age*. Although few had used HIV self-testing, all seven HIV self-testing users reported ease of use and four had tested with their partner. One participant who tested with his partner expressed: “Yes, I have even seen and received the test kits and tested myself at home with my wite, we even exchanged the test kits to see each other’s result.” *Beach 2; <35 years of age*.

### Perceived barriers to HIV self-testing use

Potential barriers to men’s HIV self-testing use included their fear of receiving a positive result, fear of stigma if a kit was discovered, and being unsure of how to use and correctly interpret the HIV self-testing kit results. Fear of a positive result was described as psychological distress if one has a seropositive result while alone rather than in the presence of an HIV counselor, as indicated in this example by a participant: “If I for instance test myself when I was not expecting the test to show that I am HIV positive…[I] may have a lot of stress for a number of days.” *Beach 3; <35 years of age*. Concerns around stigma encompassed worries that giving a HIV self-testing kit to someone or seeing someone with a HIV self-testing kit may lead others to jump to conclusions and believe that the person has HIV, as expressed by this participant: “…someone may also feel that if you give him this thing [HIV self-testing], it will be like you have judged him [HIV] positive.” *Beach 1; <35 years of age*.

Concern over the possible inability to correctly use and interpret HIV self-testing kits could be a problem that hinders use, as expressed by this participant:“It could be because if you do not know how to use it [HIV self-testing], how to start using it, you wouldn’t know. Then you take it from there [health facility], instead of using it, you are keeping it.” *Beach 2; ≥35 years of age*. Men also thought that few of their peers would go for confirmatory testing at clinics after self-testing.

### HIV self-testing distribution in the community

Nearly all Owete promoters indicated they would willingly distribute HIV self-testing to help members of their social network know their HIV status. They expressed that that these are individuals they have established relationships with and feel comfortable talking to them, as indicated by this participant: “…this is a person that I have a lot of discussions with, and I know their thoughts deeply and they also know mine, so it is easier for me to give him this thing [HIV self-testing] …” *Beach 3; <35 years of age*. Promoters stressed the importance of approaching HIV self-testing discussions strategically and thoughtfully to first garner trust and engagement through pleasantries and to assess the mood and readiness of their social network members and then to shift to more sensitive health topics, as described by this participant: “You must find a way of creating rapport with him, you make some stories then you inform him about the actual reason why you are visiting him.” *Beach 3; ≥35 years of age*. They also emphasized the importance of listening to self-testing questions from network members and the need to be trained to respond to such questions.

## Discussion

This qualitative study conducted at baseline of an intervention trial found that although few Kenyan fishermen had ever used HIV self-testing, there was generally a high level of awareness of its utility and positive perceptions of HIV self-testing. The potential benefits of HIV self-testing stemmed from the privacy afforded by testing at home, and convenience of HIV self-testing rather than having to travel to a health facility for testing, and the perceived simplicity of the method. These benefits align with those noted in other studies on HIV self-testing in SSA, and underscore that self-testing addresses many of the HIV testing barriers associated with testing at facilities (25, 44-47). The fear of having privacy compromised, feeling stigmatized, and needing to leave work are concerns with facility testing that can be mitigated with HIVST (6, 25, 32, 47, 48). This high acceptance and the perceived benefits of HIV self-testing have been confirmed in a systematic review among men in SSA and among other populations (29, 48-51). HIV self-testing may also facilite couples’ HIV testing since it can be done privately at home. Of the few in this study who had used HIVST, about half had tested with their partner. Another study in Kenya found that couples testing was more likely with HIV self-testing (75.4%) than at the health facility (33.2%) (29). This overall positive outlook on HIV self-testing utility is promising for testing initiatives.

Men selected to act as “Promoters” of HIV testing and linkage to their known peers widely expressed willingness to distribute HIV self-test kits to other men in their close social networks. The combination of men in the fishing communities indicating their interest in HIV self-testing and their influential social network-central peers being willing to distribute HIV self-testing to them illustrates the potential of HIV self-testing as a tool to know one’s status– and suggests that a peer-based approach in which peers are already known and trusted contacts may be an ideal way to reach high-priority groups of men with HIV testing. In Tanzania, 68% of men were willing to distribute HIV self-test kits to their close friends (52) and in Uganda 82% of peers were willing to distribute to fishermen in their community (53). Leveraging existing social networks to promote testing has the potential to reverse low HIV testing uptake trends among men (52). This study also revealed the importance of topic knowledge and communication style when approaching a sensitive and stigmatized condition like HIV with peers. Distribution success among peers requires establishing trust, recognizing the right time to bring up HIV testing, and confidently understanding HIV self-testing utility through training and education.

Men perceived many HIV self-testing advantages, but also voiced concerns about usage. Understanding the HIV self-testing kit procedures, results interpretation, and the absence of counseling if the test reveals HIV positive results were important concerns reported by participants. Other studies among priority populations in SSA have noted similar apprehensions (25, 48). Innovative approaches to provide HIV counseling outside of facilities directly following HIV self-testing, such as over the phone, are needed to manage the distress that may come with a positive result. Although pre- and post-test counselling are provided through confirmatory testing at the health facility where it is routine, a gap exists at the time of HIV self-testing. Moreover, as this study and other findings demonstrate, men are reluctant to go to health facilities (4, 8, 14, 25, 54, 55). Other studies involving HIV self-testing in SSA corroborate the importance of pre- and post-test counseling with HIV self-testing (44, 50). Education and training on HIV self-testing, as well as confidential peer and clinic support, may be instrumental in addressing these barriers (44, 48).

Additionally, although broad HIV self-testing awareness was indicated, it was not complemented by knowing where to obtain HIV self-tests in the community. Few participants had ever encountered HIV self-testing kits in health facilities or pharmacies. There is a need to ensure HIV self-testing kits are widely available in health facilities, pharmacies, and markets given the interest in use. A study in Tanzania found HIV self-testing availability associated with sharp increases in HIV testing (51) while a study in South Africa found that community distribution of HIV self-testing led to significantly higher testing coverage among men (56).

Given the generally low uptake of HIV services and high HIV prevalence among fishermen in western Kenya, it is important to leverage the high acceptability of HIV self-testing, the benefits it affords (privacy, efficiency, and flexibility of when and where to use) and the willingness of socially networked individuals to engage their peers and provide them with HIV self-testing information and kits. This combination of factors has the potential to inform HIV self-testing policies and strategies and improve HIV epidemic prevention and control by increasing testing uptake among those unaware of their status and improve linkage to HIV services.

This study was subject to limitations. Participants in this study were fishermen from communities around Lake Victoria in western Kenya and may not represent the views of other populations. However, given the high HIV incidence and prevalence in this area of Kenya, the viewpoints of these fishermen are critical to understanding how to better engage this high priority population in HIV testing. It is also possible that the interviews may not have captured diverse views from fishermen. We tried to minimize this by sampling widely across ages and from three distinct beach communities. It is possible that some participants may have provided responses to please the interviewer, however our experienced interviewers were not part of the fishing community, were not care providers at clinics where fishermen may seek services, and were trained to establish an open and non-judgmental demeanor in interviews, to facilitate discussion of sensitive topics. All interviewers were also native speakers of the languages spoken in the setting. Notwithstanding these limitations, study findings offer insights to inform strategies for implementation of HIVST approaches in this high priority population and setting.

## Conclusions

While few fishermen had ever used HIV self-test kits, this study found high awareness, positive perceptions, and “promoter” willingness to use and distribute HIV self-test kits to other men. The positive outlook on HIV self-testing and social network dissemination approach among fishermen may address the persistence of low HIV testing rates among men and high HIV prevalence in the region. The “promoter” model, with known peers engaged in disseminating HIV self-testing information and services, showed promise for engaging men in testing. Future programs should seek to bolster awareness of the benefits of HIV self-testing, minimize stigma and build trust among this high-risk population.

## Figures and Tables

**Table 1:** Socio-demographic characteristics of the study participants (N=65)

Characteristics	65 (%)
Study Arm	
Control	33 (50.8)
Intervention	32 (49.2)
Age	
<35	33 (50.8)
≥35	32 (49.2)
Education	
Some primary	31 (47.7)
Completed primary	20 (30.8)
Completed secondary or higher	14 (21.5)
Marital status	
Single/widowed/divorced	11 (16.9)
Married	54 (83.1)
Beach community	
Beach 1	18 (27.7)
Beach 2	23 (35.4)
Beach 3	24 (36.9)
Monthly Income	
<10,000 KSH (~ $80)	37 (57.8)
10,000 – 20,000 KSH (~ $80-$160)	17 (26.6)
>20,000 KSH (~ $160)	10 (15.6)

## Acronyms

FGDs: Focus group discussions
HIVST: HIV self-testing
IDIs: Individual in-depth interviews
IRB: Institutional Review Board
KEMRI: Kenya Medical Research Institute, Scientific Ethics Research Unit
KSHS: Kenya Shillings
SERU: Scientific and Ethics Review Unit
SSA: Sub-Saharan Africa
UCSF: University of California San Francisco
UNAIDS: Joint United Nations Programme on HIV/AIDS
USD: US Dollar
